# Chinese Translation and Cross-Cultural Adaptation of the Return-to-Work Self-Efficacy Scale among Chinese Female Breast Cancer Survivors

**DOI:** 10.3390/ijerph20054225

**Published:** 2023-02-27

**Authors:** Andy S. K. Cheng, Suki Lee, Nadia Li, Sammi Tsang, Yingchun Zeng

**Affiliations:** 1 Department of Rehabilitation Sciences, The Hong Kong Polytechnic University, Hong Kong, China; 2 Yang Memorial Methodist Social Service, Hong Kong, China; 3 Hong Kong Lutheran Social Service, Hong Kong, China; 4 Heep Hong Society, Hong Kong, China; 5 School of Medicine, Zhejiang University City College, Hangzhou 242332, China

**Keywords:** self-efficacy, return-to-work, breast cancer

## Abstract

Breast cancer is the leading cancer type among women globally. Since breast cancer has a high survival rate, most survivors are likely to return to work (RTW). In recent years, breast cancer cases have risen significantly in younger age groups. As self-efficacy is an important factor in the success of RTW, this study performed a translation and cross-cultural adaptation of the Chinese version of the Return-To-Work Self-Efficacy Scale (CRTWSE-19) and examined its psychometric properties in patients with breast cancer. This validation study followed standard guidelines, including forward translation, back translation, cross-cultural adaptation, and psychometric testing. The results of this study show that the CRTWSE-19 met reliability standards, including high internal reliability for the total scores and subscales. An exploratory factor analysis of 19 items extracted 3 factors showing consistency with the original version of the RTWSE-19. Criterion validity was demonstrated by comparing subdomains with the Fear of Cancer Recurrence Inventory. Furthermore, the known-group validity was studied by comparing mean scores among the unemployed group and the employed group. We conclude that the CRTWSE-19 has very good screening accuracy and is able to discriminate between working and unemployed populations. It can facilitate health care professionals in triaging, planning, and evaluating interventions in clinical practice.

## 1. Introduction

In 2020, there was an estimated 19.3 million new cancer cases worldwide [1]. Of those, 24% were in China, and breast cancer was the leading type of cancer [2]. Approximately 70% of the diagnosed breast cancer cases were in working-age adults [3]. Progress in early cancer detection and treatment has led to a significant decrease in cancer mortality rates, raising the overall five-year survival rate for all cancers to 67% [1]. The five-year survival rate for cancer patients at all stages of the disease, even advanced ones, is now over 90% [4]. Likewise, in Hong Kong (HK), female breast cancer was persistently the leading type of cancer amongst women aged 20 to 64 in the period of 2009 to 2017 [5]. In 2017, the crude incidence rate of breast cancer per 100,000 people was 109.3, but its crude mortality rate was only 18 [6]. The high incidence but low mortality of breast cancer patients reflects medical advances that permit earlier diagnoses and more effective treatments. Cancer is now considered a major type of non-communicable chronic disease.

Due to the high survival rate, and the young age of survivors, the prognosis is good for many breast cancer survivors (BCS) and return-to-work (RTW) is important for them [7]. However, the RTW rate among BCS has remained low: only 30.3% and 60.4% of BCS survivors returned to work within one and two years, respectively, meaning that nearly 40% of them were still unemployed two years after being diagnosed [8]. In China, Hou et al. conducted a cross-sectional study among 192 BCS and found that 21.35% of survivors in China returned to work after their primary cancer treatment [9]. A similar RTW rate was found by Li et al. in a retrospective cohort study among 396 BCS. The overall RTW rates at 12 and 36 months following cancer treatment were 24.4% and 32.7%, respectively [10]. BCS face similar workplace challenges in China to those observed in other countries [11].

Research has indicated that the success of RTW is influenced by a variety of individual and psychosocial factors such as age, socioeconomic status, self-efficacy, the fear of cancer recurrence, and social support [12]. All these factors are interrelated, and the most influential factor is self-efficacy. For example, self-efficacy is a crucial predictive factor for RTW, the success of RTW is highly affected by the fear of cancer recurrence [13], and there are significant associations between the fear of cancer recurrence, an individual’s RTW self-efficacy, and the success of RTW [14].

Self-efficacy (SE) is one’s belief in their capability to achieve specific goals through the performance of certain actions [15]. It has been recognized as an important psychological driver that can enhance RTW, although research about the role of SE in the RTW of cancer patients is limited [16]. Relevant studies on Asian populations have suggested a positive association between SE and RTW; however, a generally low level of SE in cancer patients has been discovered. Specifically, qualitative studies of Chinese breast cancer patients [9] and Singaporean head and neck cancer survivors [17] identified the phenomenon of low SE among those who did not RTW. A low level of SE reflects not only actual impaired working capability in cancer patients but also their anxiety and fear about various stressors in the workplace.

When applying the self-efficacy theory to RTW, it can be regarded as an individual’s belief in their capability of RTW. As self-efficacy is an important predictive factor for RTW, the implication is that a higher level of self-efficacy leads to a higher chance of RTW [14,18]. To assess the self-efficacy of RTW, the Return-To-Work Self-Efficacy Scale (RTWSE-19) can be adopted. The RTWSE-19 is a self-report questionnaire developed in 2011 consisting of 19 items used to assess the RTW self-efficacy of workers in resuming normal job responsibilities and predicting the success of RTW [19]. The RTWSE-19 has been adapted and validated for musculoskeletal and mental disorders [18,19,20]. In 2021, it was adapted into a Danish version for employees with cancer [21]. Unfortunately, this Danish version cannot be applied directly to the Chinese population, as it may lead to misinterpretation of the items. Both the reliability and validity of an assessment tool must be examined in the population in which it will be used in order to ensure that its psychometric properties will not be changed [22] and will be free from cultural difference between different countries. Therefore, this study aims to conduct translation and cross-cultural adaptation, testing for reliability, and psychometric testing with the Chinese version of the Return-To-Work Self-Efficacy Scale (CRTWSE-19) in breast cancer patients, so that it can become a valid and reliable measurement tool that can be used to measure self-efficacy regarding RTW among employees with cancer in Chinese communities.

## 2. Materials and Methods

### 2.1. Translation and Cross-Cultural Adaptation Processes

As mentioned above, the original version of the RTWSE-19 was used in the current study. The translation process was conducted independently at the Centre for Translation Studies, Department of Chinese and Bilingual Studies (CBS) of the Hong Kong Polytechnic University (PolyU), by a bilingual consultant whose native language was Chinese and who had no medical background and was unaware of the study proposal. It was divided into 3 steps: forward translation from the original English version into the Chinese version (step 1); back translation from the translated Chinese version from step 1 into the English version again (step 2); and confirmation by the original author of the RTWSE-19 (step 3). After confirming the content of the CRTWSE-19 with the authors, a preliminary version of the CRTWSE-19 was developed.

Three focus group discussions were carried out for the cross-cultural adaptation process and to evaluate the content validity of the preliminary version of the CRTWSE-19. The first focus group consisted of 5 experts from breast cancer teams, including 2 Breast Nurses, 2 Medical Officers, and 1 Occupational Therapist. The second focus group consisted of 5 health care staff who used breast cancer instruments frequently, including 1 Surgical Nurse in a breast cancer specialty ward, 2 Social Workers in community settings, and 2 Occupational Therapist Assistants in hospital settings. Last but not least, the third focus group consisted of 5 BCS. Members of these focus groups evaluated the relevance, representativeness, and understandability of each item in the CRTWSE-19 using a 4-point ordinal scale: 1 = not relevant/representative/understandable; 2 = somewhat relevant/representative/understandable; 3 = quite relevant/representative/understandable; and 4 = highly relevant/representative/understandable.

**Figure 1:**
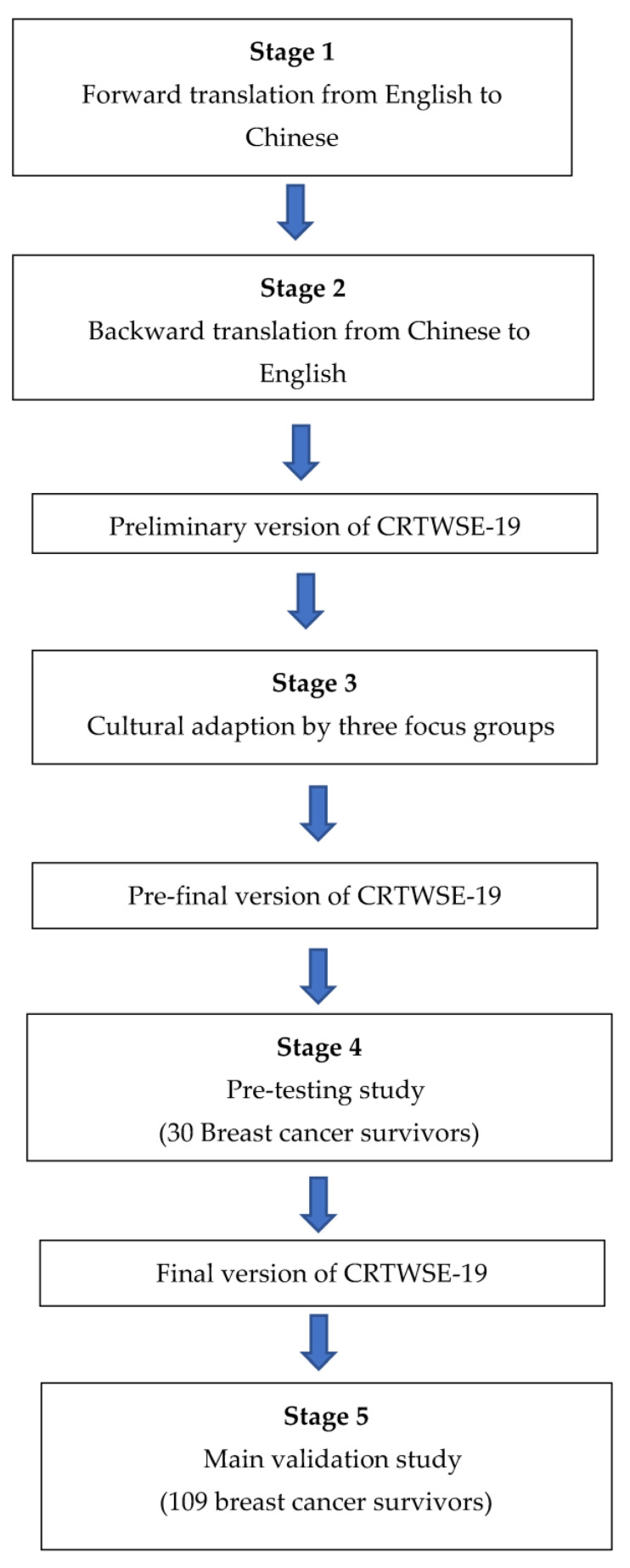
Flowchart of the translation and cross-cultural adaptation of the CRTWSE-19 (Chinese version of The Return-To-Work Self-Efficacy Scale) for breast cancer survivors.

The flowchart of the translation and cross-cultural adaptation of the CRTWSE-19 for patients with breast cancer is shown in Figure 1. 

### 2.2. Study Participants

Participants were Chinese adult women diagnosed with breast cancer. The inclusion criteria were: (1) female; (2) aged between 20 and 60 (the normal retirement age in China is 60 years old); (3) stage I–III breast cancer; and (4) completion of the primary treatment for breast cancer at least 24 months prior to the study. On the other hand, the exclusion criteria were: (1) a medical history of any psychiatric disorders; (2) an inability to provide voluntary consent; and (3) insufficient educational literacy to read and understand simple Chinese questions.

### 2.3. Outcome Measure

Based on the literature review mentioned above, self-efficacy and the fear of recurrence were proven to be the crucial inter-correlated factors affecting the success of RTW.

#### 2.3.1. Measure of the RTWSE-19

The RTWSE-19 is an English 10-point Likert scale (1 = not at all certain, 10 = completely certain) and self-report questionnaire assessing the RTW self-efficacy of workers in resuming normal job responsibilities. It has 19 items with an internal consistency of over 0.8 and 3 subscales: meeting job demands (a sample item is: “meet expectations for job performance”); modifying job tasks (a sample item is: “reduce your physical workload”); and communicating needs to others (a sample item is: “discuss openly with your supervisor things that may contribute to your discomfort”). Supplementary Materials Table S1 is the original English version of the RTWSE-19. It has adequate reliability and validity and has been adapted and validated for various clinical conditions including both musculoskeletal and mental disorders [18,20].

#### 2.3.2. Measure of the Fear of Cancer Recurrence Inventory (FCRI)

The FCRI is a validated multidimensional self-report scale for assessing the fear of cancer recurrence. It comprises 42 questions with 7 subdomains of the fear of cancer recurrence components: triggers (a sample item is: “conversations about cancer or illness in general”); severity (a sample item is: “in your opinion, what is your risk of having a cancer recurrence”); psychological distress (a sample item is: “frustration, anger or outrage”); functioning impairments (a sample item is: “my ability to make future plans or set life goals”); insight (a sample item is: “I think that I worry more about the PCR than other people who have diagnoses of cancer”); reassurance (a sample item is: “I go to the hospital or clinic for an examination”); and coping strategies (a sample item is: “I try to replace this thought with a more pleasant one”). Each item of the FCRI is rated on a 5-point Likert scale (0 = not at all or never, 4 = a great deal or all the time). Excellent internal consistency, test–retest reliability, face validity, content validity, and construct validity have been demonstrated [23]. The English version of the FCRI was translated and validated in 2016, and its internal consistency and test–retest reliability of total scales and subscales were good [24].

### 2.4. Data Collection

#### 2.4.1. Pre-Testing Study

Thirty participants were recruited to test the reliability of the pre-final version of the CRTWSE-19 in May 2018. Participants were required to fill in the questionnaire immediately, with three research investigators present to facilitate the process. The whole session was conducted in around 30 min, including screening for suitable participants and explaining the research purpose and consent. After submitting the questionnaire, every participant received a blank printed pre-final version of the CRTWSE-19 and a stamped addressed envelope. They were asked to fill in the same questionnaire seven days later and return it to PolyU by mail. Both the internal reliability and test–retest reliability were tested at this stage. The final version of the CRTWSE-19 (Supplementary Materials Table S2) was confirmed after testing the test–retest reliability.

#### 2.4.2. Main Validation Study

The final version of the CRTWSE-19 was administered in psychometric testing for construct validity, criterion validity, and known group validity. The FCRI was adopted to evaluate the validity of the final version. All participants were asked to fill in the final versions of the CRTWSE-19 and the FCRI, and informed consent was given by the eligible participants for this main validation study.

### 2.5. Data Analysis

In the tests for internal reliability, coefficient alphas were analyzed to give the total score of the CRTWSE-19 and the 3 subscales. Following the standard of cultural adaptation validation, a Cronbach alpha coefficient higher than 0.7 is considered satisfactory [25]. Additionally, intraclass correlation coefficients (ICC) were used to demonstrate test–retest reliability, with ICC values between 0.75 and 0.9 indicating good reliability [26]. The content validity was tested by evaluating the relevance, representativeness, and understandability of each item, with items considered as reasonably relevant if the content validity of both the individual items (I-CVI) and overall scale (S-CVI) was larger than 0.78 [27]. Exploratory factor analysis was used to determine construct validity by individual item analysis and factor analysis (principal component analysis with varimax rotation) to verify the numbers of factors. Furthermore, criterion validity was determined by comparing correlations between the subscales of the CRTWSE-19 and the domains of the FCRI, with the correlations indicating that some similar items with conceptual overlapping in specific areas were present in both instruments. Lastly, the known group validity was tested by comparing the mean scores of the CRTWSE-19 of the unemployed and employed groups, with the mean score considered acceptable if the CRTWSE-19 of the unemployed group was significantly lower than that of the employed group. Finally, receiver operating curve (ROC) analyses were carried out to evaluate the screening accuracy of this scale in discriminating between those who can RTW or not. Calculating sensitivity and specificity for the CRTWSE-19 scale requires a cutoff score. Youden’s index [28] was used to choose an optimal cutoff score. All statistical analyses were performed using the SPSS program, version 23.0 for Windows, and the significance level was set to *p* < 0.05.

### 2.6. Ethics

Ethical approval was obtained through the Institutional Review Board of PolyU before the commencement of the study. Ethical approval of the research study conducted in the North District Hospital (NDH) under the New Territories East Cluster of Hospital Authority was successfully obtained via the Joint Chinese University of Hong KONG—New Territories East Cluster Clinical Research Ethics Committee (joint CUHK-NTEC CREC). Informed and written consent was explained to, and given by, the participants from the NDH.

## 3. Results

### 3.1. Descriptive Data

A total of 139 participants were recruited in this study. Table 1 shows the sociodemographic characteristics of the breast cancer patients participating in the pre-testing and main validation studies. The majority were diagnosed with early-stage breast cancer, and more than half had undergone combined surgical, chemotherapy and radiotherapy treatment. In terms of occupational status, more than half of the BCS were employed: 43.3% of the working group had returned to their previous work in the pre-testing stage, and 32.1% of the working group had returned to their previous work (from before disease onset) in the main validation study (Table 1).

### 3.2. Reliability

The CRTWSE-19 demonstrated high internal reliability, with its Cronbach alpha scoring 0.97 for the pre-testing study and 0.93 for the main validation study. The Cronbach alpha of the three subscales of the CRTWSE-19 (“Meeting job demands”, “Modifying job tasks” and “Communicating needs to others”) scored 0.97, 0.96, and 0.92 in the pre-testing study, and 0.89, 0.92, and 0.87 in the main validation study, respectively. As they were all above 0.7, this result was considered acceptable [25]. In addition, there was a high test–retest reliability of the total score of the CRTWSE-19 (ICC = 0.89, subscales of ICC = 0.84, 0.92, and 0.88).

### 3.3. Validity

#### 3.3.1. Content Validity

Content validity was conducted during the translation and cultural adaptation processes. Three focus groups were defined: (1) experts in breast cancer, (2) health care staff who use breast cancer instruments frequently, and (3) BCS. For focus groups 1 and 2, the I-CVI and S-CVI were calculated. S-CVI/Avg = 0.9, and all I-CVIs scored above 0.78 except for items 7 and 14. Overall, a value above 0.78 was considered acceptable based on the previous literature [27]. Based on the comments of the focus group members, item 7 was revised by adding the word “cancer recurrence” to the end of the question. Item 14 was revised by changing the word “extremity” to “body”. The understandability of the CRTWSE-19 was good according to the three focus groups. As well as the focus groups, a bilingual consultant’s professional advice was sought, but no significant changes were required. The contents of both the CRTWSE-19 and the FCRI were accepted after the amendments were made.

#### 3.3.2. Construct Validity

Exploratory factor analysis of the 19 items extracted 3 factors with eigenvalues >1, with nearly 89% of factor solutions explained by variances. Varimax rotation was used to identify the contributing items in corresponding factors. See Table 2. The original version of the RTWSE-19 labeled the three factors as “Meeting job demands” (seven items), “Modifying job tasks” (seven items), and “Communicating needs to others” (five items), [19]. Our findings showed that the CRTWSE-19 demonstrated high construct validity when compared with the original version of the RTWSE-19 and the verified conceptual subdomains (Table 2).

#### 3.3.3. Criterion Validity

Criterion validity was determined by assessing the correlation between the CRTWSE-19 and the FCRI. The correlation between the subdomains of the FCRI and the subscales of the CRTWSE-19 was compared. A moderate negative correlation was demonstrated between the “functioning impairments” subdomain in the FCRI and the subscales of “meeting job demands” (*r* = −0.575, *p* < 0.01), “modifying job tasks” (*r* = −0.481, *p* < 0.01) and “communicating needs to others” in the CRTWSE-19 (*r* = −0.556, *p* < 0.01). In addition to this, a moderate negative correlation was shown between the “insight” subdomain in the FCRI and the “communicating needs to others” subscale in the CRTWSE-19 (*r* = −0.475, *p* < 0.01). Overall, there was a slight negative correlation between the total scores of the FCRI and the CRTWSE-19 (*r* = −0.235, *p* < 0.01).

#### 3.3.4. Known Group Validity

The mean scores of the unemployed group (5.76) and employed group (7.44) were calculated and compared using the *t*-test. A mean difference (−1.68, *p* < 0.01) was demonstrated, indicating that the mean score of the unemployed group was 1.68 lower than the mean score of the employed group.

#### 3.3.5. Screening Accuracy

**Figure 2:**
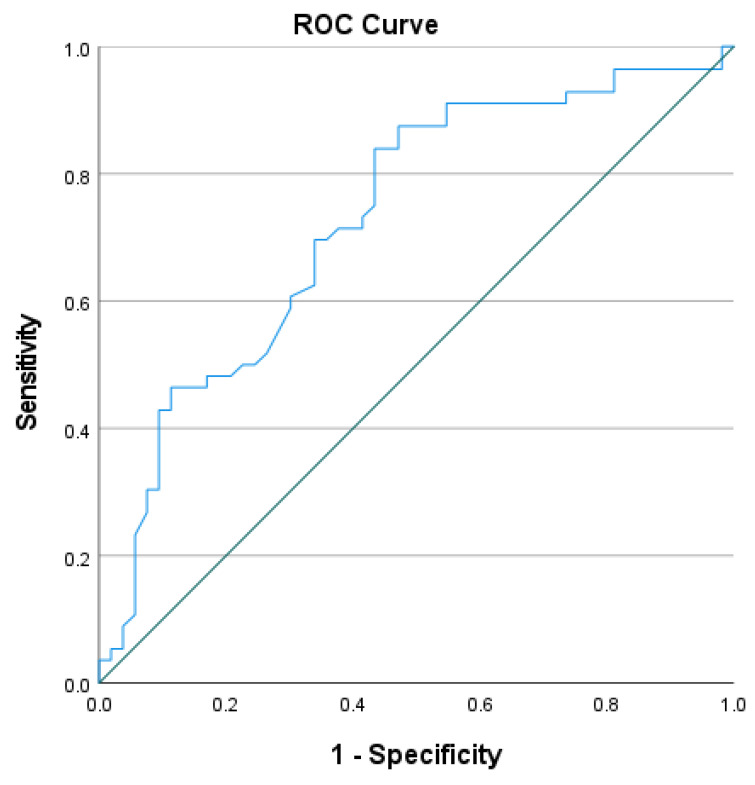
ROC curve of the CRTWSE-19 total mean score and RTW of breast cancer survivors (n = 109).

Figure 2 presents the ROC curve associated with the total mean score of the CRTWSE-19. The area under the curve serves as an overall measure of discrimination between those who can RTW or not. An area of 1 refers to perfect discrimination, whereas an area of 0.5 refers to a test that failed to discriminate. According to Hosmer and Lemeshow [29], an area of at least 0.70 indicates acceptable discrimination and an area of at least 0.80 indicates excellent discrimination. Overall, the area under the curve was 0.729 (*p* < 0.0001, 95% CI 0.633–0.824) at a cutoff score of 6.24 with a sensitivity of 83.9% and specificity of 56.6% (Figure 2).

## 4. Discussion

Overall, the CRTWSE-19 has acceptable internal and test–retest reliability and has good content validity, constructed validity, and screening accuracy. The findings of this study indicate that the CRTWSE-19 is a valid instrument in the context of breast cancer, can discriminate between the working and the unemployed populations, and is indirectly, proportionally correlated with the fear of cancer recurrence, especially in terms of functioning impairments and insight. The original version was divided into three score ranges (1–5 = low self-efficacy; 5–7.5 = medium self-efficacy; and 7.5–10 = high self-efficacy) [18]. Our study provides a clearer predictive value of the CRTWSE-19 to assess the probability that a breast cancer survivor will RTW. Therefore, a BCS with a CRTWSE-19 mean score lower than 6.24 would have a problem in RTW. Consequently, the CRTWSE-19 can provide a more effective and efficient triage system that can be developed and implemented in a clinical setting.

The CRTWSE-19 may serve as the first Chinese self-reported instrument used to assess the RTW self-efficacy of BCS in the context of the success of RTW in the Chinese population. With its cross-cultural adaptation, it succeeded in maintaining its original meaning while including characteristics peculiar to the Chinese community. It can be used in various clinical settings, such as hospitals, non-governmental organizations, vocational rehabilitation training centers, and private clinics. As mentioned before, RTW is attributed to different factors, including physical and psychological factors that are interrelated and influence each other. Thus, both physical (e.g., clinical oncology and work rehabilitation) and psychiatric settings could use this instrument and would benefit from it. Furthermore, individuals can fill in the questionnaire by themselves without translation by health care professionals, making it more convenient and efficient.

As the CRTWSE-19 is divided into three factors, it is possible to evaluate and reflect on which of these factors most affect BCS in terms of RTW. Health care professionals may provide corresponding interventions for BCS based on their CRTWSE-19 scores. Overall, enhancing RTW self-efficacy and lifestyle redesign programs are recommended for low-scoring patients [30], while job matching, simulated work training, and job-hunting opportunities are suitable for high-scoring patients. It is worth mentioning that work-related factors, such as the type of job, sector of activity or job demand, level of income [31], the prevailing stigma, and the presence/absence of discrimination toward cancer patients [32], have also been suggested as factors that affect the RTW of cancer survivors in Asian countries. However, previous systematic reviews showed that RTW is dependent on the context, which includes perspectives that go beyond health care. Some workplace factors have been found to be negatively associated with RTW. RTW would be very difficult, or even impossible, in a non-supportive, rigid, and competitive work culture [33]. In addition, any major workplace changes after a survivor’s diagnosis and treatment, such as corporate restructuring or personnel changes, also make RTW more difficult [34]. On the other hand, most cancer survivors describe how long-term symptoms and side effects arising from cancer treatment can impact their work ability over a long period of time. Therefore, they require flexibility and adjustments on their part and also on the part of their colleagues and employers. Last but not least, workplace policies, procedures, culture, and resources have emerged as major factors impacting the negative and positive experiences of cancer survivors in RTW [35]. Therefore, effective RTW interventions will likely involve a multifactorial approach, moving beyond the management of symptoms and side effects to addressing psychosocial, systemic, and environmental issues [36]. The effectiveness of such interventions can be evaluated using the CRTWSE-19.

## 5. Conclusions

Because of medical advances facilitating earlier diagnosis and more effective treatments, cancer is now considered as a major type of non-communicable chronic disease. However, cancer is still one of the major diseases which impair an individual’s normal life. Many cancer patients have to leave their jobs temporarily during cancer treatment. Unfortunately, some patients are forced to leave their jobs permanently because of residual physical and psychosocial complications that prevent them from engaging in their occupational activities. Individuals who are unable to RTW can impose a burden on their families and society. In addition to the financial costs associated with loss of work due to cancer, the inability to return to work also has some negative psychological consequences for cancer survivors. Consequently, it not only decreases their life expectancy and motivation but also causes anxiety for their families. Therefore, promoting RTW among cancer survivors is relevant and important for the full recovery of cancer patients. This study translated and cross-culturally adapted the instrument of the CRTWSE-19 to measure the RTW self-efficacy of BCS. The CRTWSE-19 can be used in various clinical settings, including hospitals, non-governmental organizations, and private clinics. Both psychiatric and physical settings (e.g., clinical oncology and work rehabilitation) can benefit from it. The CRTWSE-19 can facilitate health care professionals in triaging, planning, and evaluating interventions in clinical practice.

## Figures and Tables

**Table 1:** Characteristics of breast cancer survivors participating in the pre-testing and main validation studies.

Variables	Pre-Testing Study (N = 30)	Main Validation Study (N = 109)
	n (%)	
Gender (female)	30 (100)	109 (100)
Age (years)		
<60	21 (70.0)	67 (61.5)
≥60	9 (30.0)	42 (38.5)
Highest education level		
Primary school	6 (20.0)	35 (32.1)
Secondary school	21 (70.0)	59 (54.1)
College or above	3 (10.0)	15 (13.8)
Employment status		
Unemployed	12 (40.0)	53 (48.6)
Full-time job	17 (56.7)	56 (51.4)
Others	1 (3.3)	
Disease stage		
Early stage	23 (76.6)	59 54.1)
Middle stage	5 (16.7)	44 (40.4)
Advanced stage	2 (6.7)	6 (5.5)
Treatment types		
Single type such as surgery or chemotherapy only	6 (20.0)	12 (11.1)
Combined types of treatment	24 (80.0)	97 (88.9)

**Table 2:** Rotated component matrix of the CRTWSE-19, assessed by factor analysis.

CRTWSE-19 Item	Component		
	1	2	3
Item 1	0.310	**0.816**	0.288
Item 2	**0.844**	0.343	0.305
Item 3	0.345	**0.789**	0.365
Item 4	0.390	0.332	**0.783**
Item 5	**0.825**	0.363	0.340
Item 6	**0.795**	0.329	0.404
Item 7	0.446	**0.749**	0.318
Item 8	0.399	0.308	**0.783**
Item 9	**0.821**	0.366	0.314
Item 10	0.431	**0.755**	0.404
Item 11	0.322	0.330	**0.817**
Item 12	0.308	**0.853**	0.269
Item 13	**0.791**	0.421	0.343
Item 14	0.430	**0.737**	0.393
Item 15	**0.716**	0.456	0.418
Item 16	0.304	0.375	**0.799**
Item 17	0.420	0.432	**0.740**
Item 18	**0.754**	0.380	0.382
Item 19	0.440	**0.701**	0.453

Extraction method: principal component analysis. Rotation method: varimax with Kaiser normalization. Bold numbers have the highest factor loadings which fit best into different component.

## Data Availability

The data presented in this study are available on request from the corresponding author due to privacy reason.

## Supplementary Materials

The following supporting information can be downloaded at: https://www.mdpi.com/article/10.3390/ijerph20054225/s1, Table S1. English version of RTWSE-19. Table S2. Chinese version of RTWSE-19 adapted for cancer patient.
