# Supplementary material for: Chinese Translation and Cross-Cultural Adaptation of the Return-to-Work Self-Efficacy Scale among Chinese Female Breast Cancer Survivors
Source: Int J Environ Res Public Health. 2023 Feb 27;20(5):4225. doi: 10.3390/ijerph20054225 (PMC10001523; doi:10.3390/ijerph20054225)
Supplement: Supplementary file 1 [file ijerph-20-04225-s001.zip › ijerph-2173161-supplementary.pdf]

Table S1. English version of RTWSE-19.

We would like to know how confident you are in doing certain activities if you were at work today. For each of the following questions, please circle the number that corresponds to your confidence that you could do the task at the present time:

How confident are you that you could…	Not at all confident (1)									Totally confident (10)
1. Suggest to your supervisor ways to change your work to reduce discomfort?	1	2	3	4	5	6	7	8	9	10
2. Fulfill all of your duties and responsibilities?	1	2	3	4	5	6	7	8	9	10
3. Change they type of work activities you do to reduce discomfort?	1	2	3	4	5	6	7	8	9	10
4. Explain any physical limitations you may have to your co-workers?	1	2	3	4	5	6	7	8	9	10
5. Meet expectations for job performance?	1	2	3	4	5	6	7	8	9	10
6. Perform most of your daily activities at work?	1	2	3	4	5	6	7	8	9	10
7. Avoid re-injury?	1	2	3	4	5	6	7	8	9	10
8. Get co-workers to help you with activities that might cause discomfort?	1	2	3	4	5	6	7	8	9	10
9. Keep up with the pace at work?	1	2	3	4	5	6	7	8	9	10
10. Modify the way you work to reduce discomfort?	1	2	3	4	5	6	7	8	9	10
11. Get emotional support from co-workers (such as listening or talking about your problem)?	1	2	3	4	5	6	7	8	9	10
12. Avoid activities that are likely to increase pain?	1	2	3	4	5	6	7	8	9	10
13. Meet your production requirements?	1	2	3	4	5	6	7	8	9	10
14. Reduce your physical workload?	1	2	3	4	5	6	7	8	9	10
15. Do everything you're trained to?	1	2	3	4	5	6	7	8	9	10
16. Describe to your supervisor the nature of your injury and your medical treatment?	1	2	3	4	5	6	7	8	9	10
17. Discuss openly with your supervisor things that may contribute to your discomfort?	1	2	3	4	5	6	7	8	9	10
18. Do your work without slowing others down?	1	2	3	4	5	6	7	8	9	10
19. Request changes in your workstation or work area to reduce discomfort?	1	2	3	4	5	6	7	8	9	10

Table S2. Chinese version of RTWSE-19 adapted for cancer patient.

說明: 假設您今天正在工作, 我們想知道您在做以下事情時有多大的信心。請就以下描述的各個情況圈出最能代表您目前可以完成該事情的信心數值。

您有多大的信心能……	完全沒信心									完全有信心
1. 向上司建議減低工作時不適的方法?	1	2	3	4	5	6	7	8	9	10
2. 完成所有職責?	1	2	3	4	5	6	7	8	9	10
3. 改變工作內容以減低不適?	1	2	3	4	5	6	7	8	9	10
4. 向同事解釋您可能會有的身體限制?	1	2	3	4	5	6	7	8	9	10
5. 達到工作表現的標準?	1	2	3	4	5	6	7	8	9	10
6. 完成日常大部分的工作?	1	2	3	4	5	6	7	8	9	10
7. 避免再次受傷或癌症復發?	1	2	3	4	5	6	7	8	9	10
8. 在可能會引起不適的工作上請同事幫忙?	1	2	3	4	5	6	7	8	9	10
9. 保持工作進度?	1	2	3	4	5	6	7	8	9	10
10. 調整工作方式以減少不適?	1	2	3	4	5	6	7	8	9	10
11. 得到同事精神上的支持(例如聆聽或與同事傾談您的困難)?	1	2	3	4	5	6	7	8	9	10
12. 避免可能會增加疼痛的工作?	1	2	3	4	5	6	7	8	9	10
13. 達到生產力要求?	1	2	3	4	5	6	7	8	9	10
14. 減低工作對您身體的負荷?	1	2	3	4	5	6	7	8	9	10
15. 做到所有您曾被訓練的任務?	1	2	3	4	5	6	7	8	9	10
16. 向上司說明妳的癌症性質和治療?	1	2	3	4	5	6	7	8	9	10
17. 向上司坦誠討論可能引致您不適的事情?	1	2	3	4	5	6	7	8	9	10
18. 做到您的工作而不會拖慢別人的進度?	1	2	3	4	5	6	7	8	9	10
19. 請求改變工作場所和一些設施以減低不適?	1	2	3	4	5	6	7	8	9	10
